# Trained immunity in atherosclerosis: plasticity, metabolic-vascular axis, and AI-driven precision remodeling

**DOI:** 10.3389/fimmu.2025.1669796

**Published:** 2025-10-10

**Authors:** Bing Zhao, Jiayang Wan, Huifen Zhou, Jiehong Yang, Haitong Wan

**Affiliations:** ^1^ College of Chinese Medicine for Cardiovascular-Cranial Disease, Zhejiang Chinese Medical University, Hangzhou, China; ^2^ Zhejiang Key Laboratory of Chinese Medicine for Cardiovascular and Cerebrovascular Disease, Zhejiang Chinese Medical University, Hangzhou, China; ^3^ The First Affiliated Hospital, Zhejiang University School of Medicine, Hangzhou, China; ^4^ Academy of Chinese Medical Sciences, Henan University of Chinese Medicine, Zhengzhou, China

**Keywords:** trained immunity, atherosclerosis, metabolic-vascular axis, immune plasticity, AI-driven precision medicine

## Abstract

Chronic inflammation linked to atherosclerosis is closely related to a trained immunoregulatory network. Traditional studies primarily focus on the pro-inflammatory memory of monocytes, they frequently neglect important aspects such as the cell’s plasticity, interactions between different organs, and the dynamic regulation of the metabolism-vascular axis. This review presents four novel frameworks, including the trained immunity plasticity spectrum model. It demonstrates how monocytes maintain a dynamic balance between pro-inflammatory, tolerogenic, and anti-inflammatory phenotypes, regulated by mTOR/AMPK signaling and competitive histone modifications. The trained immunity–metabolism–vascular axis shows that metabolic disorders can change the way immune memory is formed. They achieve this by modifying the vascular microenvironment through epigenetic changes, exosomes, and products of mitochondrial stress. The cross-organ trained immunity framework reveals how remote epigenetic communication between the bone marrow, gut, and liver influences the development of monocytes. Finally, dynamic immune reprogramming integrates CRISPR-based epigenetic editing, metabolism-focused interventions, and AI-driven multi-omics predictions. This approach signifies a major transition from simply alleviating symptoms to accurately reshaping immune memory. This review reinterprets the immunometabolic mechanisms of atherosclerosis. It also lays the foundation for personalized therapies enhanced by AI and explores new interdisciplinary research avenues.

## 1 Background

Atherosclerosis is a chronic inflammatory condition marked by lipid-related issues in blood vessels (1). It arises from inappropriate responses of the innate immune system (2). Monocytes and macrophages play key roles in the development and worsening of plaque in the arteries (3); they cause persistent inflammation by stimulating oxidized low-density lipoprotein (oxLDL) (4), releasing cytokines (5), and forming foam cells (6). Even with lipid-lowering treatments, blood vessel inflammation continues (7), exposing a crucial gap in our understanding: the factors behind the prolonged activation of innate immune cells extend beyond traditional inflammatory processes (8).

The discovery of trained immunity (9, 10), has significantly changed our understanding of chronic inflammatory diseases. This term refers to the reprogramming of innate immune cells, allowing them to exhibit memory-like responses due to alterations in their epigenetic and metabolic profiles (11). Initially identified in the context of infections, trained immunity is now recognized as a factor in atherosclerosis (12), where triggers such as oxLDL and hyperglycemia lead to lasting proinflammatory changes in monocytes (13). These changes happen through mechanisms such as histone modifications, specifically H3K4me3 (14), and metabolic shifts like increased glycolysis (15). Most current research focuses on the pro-inflammatory aspects of trained immunity, often ignoring its flexibility and the wider regulatory networks involved (16). Bekkering (17) and colleagues showed that oxLDL can cause epigenetic changes in monocytes. However, the potential for trained immunity to also play tolerogenic or reparative roles has not been fully explored. Furthermore, the interactions between various organs, including the bone marrow’s role in blood cell production (18) and the effects of gut microbiota metabolites (19), have not been sufficiently explored in relation to trained immunity. Although evidence indicates that systemic metabolic issues may lead to vascular inflammation (20), this topic is still underexplored.

**Figure 1:**
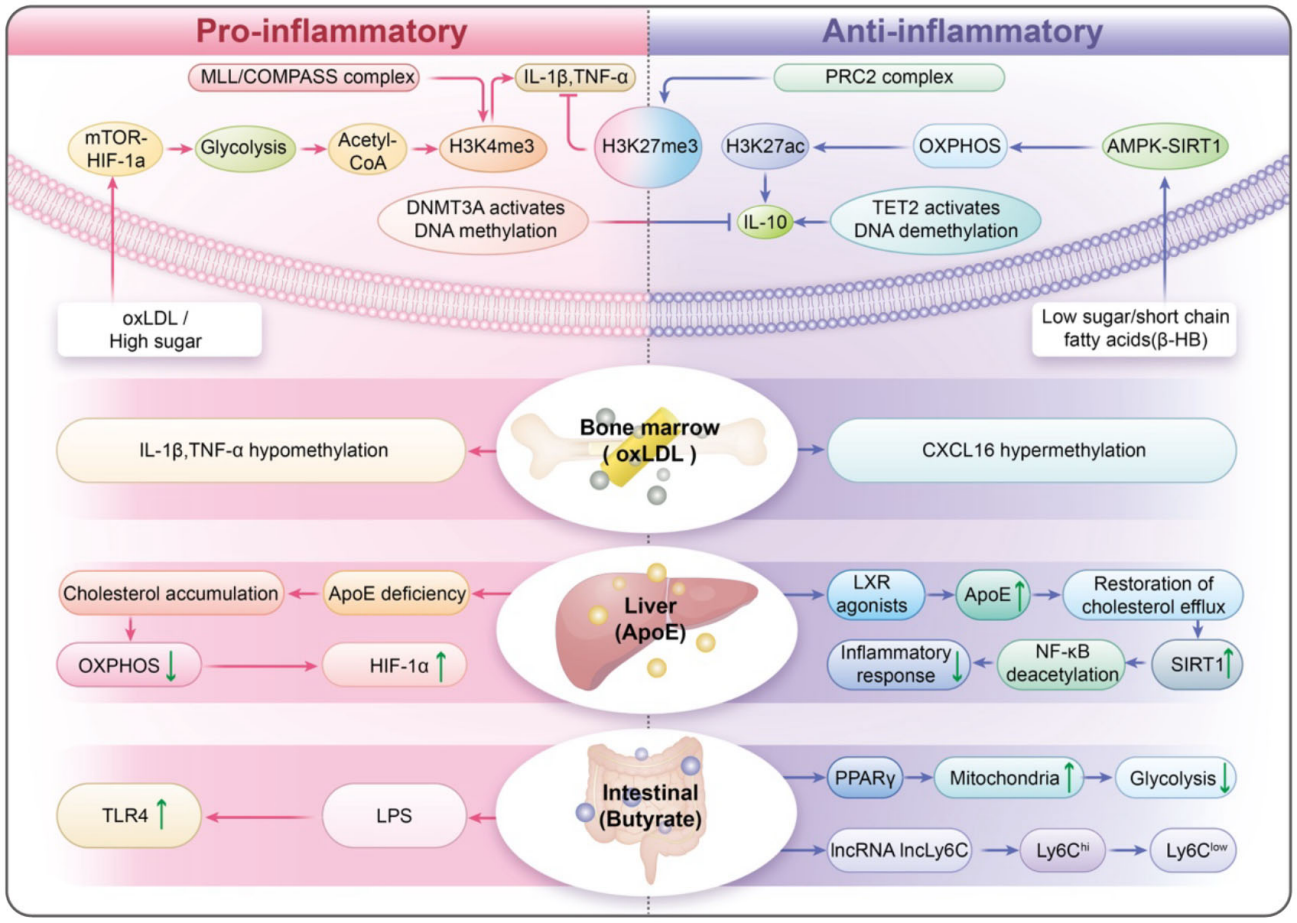
Trained Immunity Plasticity Spectrum (TIPS): A Dynamic Equilibrium of Immune Memory. The Cross-Organ Trained Immunity (COTI): highlights the role of bone marrow-derived hematopoietic stem cells, metabolites from gut microbiota, like short-chain fatty acids, and apolipoproteins produced by the liver in regulating the fate of monocytes.

This review examines the limitations of our current knowledge by utilizing four interconnected frameworks. The trained immunity plasticity spectrum redefines trained immunity as a dynamic balance among pro-inflammatory, tolerogenic, and anti-inflammatory phenotypes, influenced by mTOR/AMPK signaling pathways (21) and opposing histone modifications (H3K4me3 versus H3K27me3) (22, 23). The trained immunity–metabolism–vascular axis shows how metabolic disturbances, like abnormal cholesterol synthesis and high blood sugar, can epigenetically influence monocytes (24). These disturbances also alter the vascular environment through exosomal miRNAs and signals from mitochondrial stress (25). The cross-organ trained immunity highlights the role of bone marrow-derived hematopoietic stem cells, metabolites from gut microbiota, like short-chain fatty acids (26), and apolipoproteins produced by the liver in regulating the fate of monocytes (27) (**Figure 1** **).** Dynamic immune reprogramming proposes several strategies. These include CRISPR-based epigenetic editing (28), therapies that target metabolism, and integrating computational multi-omics (29). Together, these approaches aim for precise modulation of trained immunity.

By integrating mechanistic insights with translational innovation, this synthesis significantly redefines atherosclerosis as an “immune-metabolic memory disorder” and encourages the field to move beyond oversimplified models. Future initiatives should use interdisciplinary strategies that include spatial multi-omics, quantum-enabled epigenomic mapping, and global collaborations to fully explore the therapeutic potential of translational innovation.

## 2 The trained immunity plasticity spectrum: from proinflammatory dominance to dynamic equilibrium

### 2.1 The proinflammatory paradigm: foundations of classical trained immunity

The classical understanding of trained immunity focuses on its role in sustaining pro-inflammatory responses in innate immune cells (30). Early research indicates that, particularly in the context of infections or β-glucan exposure (31), monocytes and macrophages can undergo significant changes in metabolism and epigenetics when exposed to inflammatory stimuli such as oxidized LDL or lipopolysaccharides (LPS) (2). This reprogramming causes an increased production of cytokines, including IL-1β, IL-6, and TNF-α, during subsequent challenges (32). The “proinflammatory-centric” model highlights mechanisms such as mTOR-HIF-1α signaling and trimethylation of histone H3 at lysine 4 (H3K4me3) at the promoters of proinflammatory genes, including IL-1β and TNF (33, 34). These mechanisms help stabilize glycolytic metabolism and enhance inflammatory memory. Although this framework established an important foundation, it fails to explain why inflammatory markers stay elevated even after the initial triggers have disappeared. It did not consider the variability in monocyte responses found in conditions such as atherosclerosis, where both proinflammatory and anti-inflammatory subsets exist in plaques (35).

### 2.2 Redefining immune memory: the TIPS model and its dynamic equilibrium

The trained immunity plasticity spectrum model offers a fresh view of trained immunity, depicting it as a dynamic range that encompasses pro-inflammatory, tolerogenic, and anti-inflammatory phenotypes. Proinflammatory trained immunity is triggered by metabolic stressors like oxLDL or high glucose levels (36). In the Ldlr−/− model, a Western diet can induce persistent training-induced immunity (NLRP3-dependent), and inflammatory memory characteristics remain even after dietary correction (37). Short-term oxLDL pretreatment induces H3K4me3 enrichment and enhances re-stimulation responses in human monocytes, resulting in long-term pro-inflammatory/pro-foam cell memory (38). This state reduces glycolysis and boosts oxidative phosphorylation while also adding repressive histone modifications (H3K27me3) to proinflammatory enhancer regions (39). In ApoE^−/−^ and AAV-PCSK9 mice fed a high-fat diet, 4-PBA-trained monocytes exhibited reduced adhesion and increased CD24 expression, among other pro-differentiation features, and achieved sustained reprogramming through inhibition of SYK–mTOR, restoration of peroxisomal homeostasis, and TOLLIP-PPARγ neddylation; Whether administered systemically or transplanted as trained monocytes, they significantly reduced plaque burden and increased collagen content, and transmitted anti-inflammatory memory via CD24 between recipient monocytes, providing direct evidence for anti-inflammatory trained immunity in an atherosclerotic context (40). In parallel, the ketone body β-hydroxybutyrate (β-HB) functions as an endogenous HDAC inhibitor, elevating histone H3 acetylation (e.g., H3K9/14ac) at immune-regulatory loci (41, 42). Consistent with this epigenetic shift, oral 3-hydroxybutyrate in ApoE^−/−^ mice reduced plaque burden and redirected monocyte–macrophage responses toward a reparative program, while complementary human ex vivo and murine data show β-HB suppresses NLRP3-dependent IL-1β/IL-18 production (43, 44). These states are dynamic and maintain a balance, influenced by metabolic and epigenetic signals that can change the fate of monocytes (45).

### 2.3 Regulatory nodes of plasticity: metabolic, epigenetic, and microenvironmental control

Metabolic regulation is essential for cellular function, and the mTOR/AMPK axis acts as a key metabolic switch (21). Under low glucose conditions, AMPK activates and phosphorylates the autophagy-initiating kinase Unc-51-like kinase 1 (ULK1), promoting autophagosome formation and cellular energy recovery (46). In contrast, when nutrients are plentiful, mTORC1 inhibits autophagy by phosphorylating ULK1 (47). The dynamic interplay between AMPK and mTORC1 facilitates cellular adaptation to metabolic stress (48). In addition, metabolites such as α-Ketoglutaric acid (α-KG) and acetyl-CoA play important roles in regulating epigenetics (49). For example, α-KG activates the TET2 enzyme to promote DNA demethylation and activate anti-inflammatory genes, such as IL-10 (50). Acetyl-CoA drives histone acetylation and helps establish a pro-inflammatory memory within the cell (51). The interaction of epigenetic modifications causes antagonism. Competing histone modifications, particularly H3K4me3 and H3K27me3, function as a chromatin “toggle switch” (52). The MLL/COMPASS complex deposits H3K4me3 at inflammatory loci, while the PRC2 complex deposits H3K27me3, silencing these genes in conditions that promote tolerance (53). Moreover, antagonism of DNMT3A with TET2 ensures that gene promoter methylation levels are under dynamic regulation and adapt to environmental changes (54). The microenvironment also plays a significant role in shaping monocyte trained immunity, influenced by various signals such as cytokines (like IFN-γ and IL-10), metabolites (including lactate and succinate), and hypoxic conditions (55). Hypoxia within plaques stabilizes HIF-1α, which amplifies proinflammatory responses associated with trained immunity (56). Microbiota-derived short-chain fatty acids—notably butyrate—act as endogenous histone deacetylase inhibitors and dampen trained-immunity induction in humans; in ApoE^−/−^ mice, SCFAs mitigate atherosclerotic inflammation via GPR43/HDAC-linked pathways (57–60).Likewise, an ApoE^-/-^ mouse model treated with orally sodium butyrate (NaB) demonstrated that butyrate derived from intestinal flora regulates Mψs polarization through the GPR43/HDAC-miRNAs axis. This regulation leads to a decrease in pro-inflammatory factors such as IL-6 and TNF-α in arterial plaques, while increasing the anti-inflammatory factor IL-10, which ultimately reduces plaque area (59).

## 3 Metabolic reprogramming and vascular axis: from epigenetic memory to therapeutic innovation

### 3.1 Metabolic derangements and epigenetic rewiring of immune memory

Metabolic disorders such as high cholesterol and high blood sugar significantly affect immune memory by changing the epigenetic characteristics of monocytes (61). In cases of hypercholesterolemia, oxLDL activates the mevalonate pathway, leading to the production of isoprenoid intermediates, such as farnesyl pyrophosphate (62, 63). By stabilizing HIF-1α and enhancing mTORC1 signaling, this process induces a metabolic shift that increases glycolytic flux (34). This transformation leads to the production of acetyl-CoA, which is utilized as a substrate by HATs (64, 65). Consequently, increased histone acetylation at inflammatory promoters sustains transcriptional memory in innate cells (66). Similarly, high blood sugar levels activate the hexosamine biosynthesis pathway (HBP), resulting in increased O-GlcNAcylation of nuclear factor κB and histones (67). This modification increases the transcription of inflammatory genes, even after glucose levels normalize. Epigenetic “scars” can remain even after metabolic disturbances have resolved. This persistence locks monocytes into a proinflammatory state, which promotes the onset and progression of antiphospholipid syndrome (APS) and AS (68, 69). For example, monocytes from APS display continuous H3K4 trimethylation at the ARID5B promoter, which plays a role in apoptosis and pyroptosis (70). This example illustrates epigenetic ‘scars’ in chronic inflammation outside atherosclerosis and is hypothesis-generating for vascular disease.

### 3.2 Metabolic-immune dialogue and vascular microenvironment remodeling

**Figure 2:**
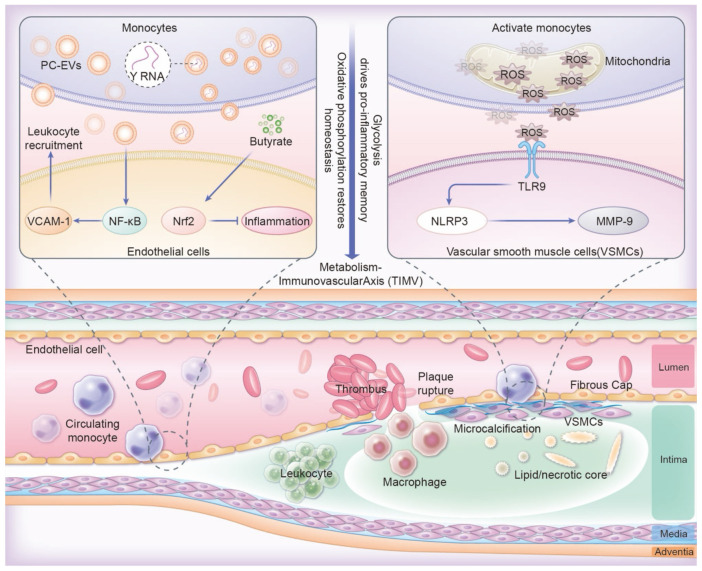
The TIMV axis links metabolism with trained immunity and vascular regulation. Pro-inflammatory PC-EVs activating NF-κB, disrupting VCAM-1 circadian control and amplifying leukocyte recruitment. Butyrate activating endothelial Nrf2 for plaque stabilization. Mitochondrial ROS in monocytes triggers TLR9/NLRP3 activation in VSMCs, driving MMP-9 secretion and collagen breakdown through macrophage infiltration, destabilization of atherosclerotic plaques.

Altered monocytes interact with vascular cells, significantly influencing the development of atherosclerotic areas. This influence occurs through the release of extracellular vesicles and signals associated with mitochondrial dysfunction (71). Proinflammatory monocytes produce PC-EVs, which activate the NF-κB pathway in endothelial cell (71–73). This activation intersects with the circadian control of adhesion molecules (e.g., VCAM-1), thereby promoting leukocyte recruitment (74). Mitochondrial dysfunction in activated monocytes triggers the release of mitochondrial DNA fragments and reactive oxygen species (ROS). These releases, in turn, activate Toll-like receptor 9 (TLR9) and the NLRP3 inflammasome in vascular smooth muscle cells (VSMCs) (75). This activation causes vascular smooth muscle cells (VSMCs) to adopt a synthetic phenotype, which is characterized by the secretion of matrix metalloproteinase-9 (MMP-9) and collagen breakdown, further destabilizing atherosclerotic plaques (76). Anti-inflammatory metabolites, such as SCFAs, mitigate these harmful effects (60, 77). For instance, butyrate directly activates Nrf2 signaling in endothelial cells via p300-mediated transcriptional activation, enhancing antioxidant defenses and endothelial function (78). This activation increases antioxidant defenses and helps stabilize plaque (**Figure 2**).

### 3.3 Future frontiers: competitive metabolite dynamics and spatial multi-omics

The intricate and unresolved complexities of metabolism-immune system interactions demand innovative strategies. Competition between metabolites is critical (79). Ketones (β-HB) and lactate influence the availability of acetyl-CoA and subsequently affect epigenetic outcomes by altering histone acetylation patterns (80). In areas of atherosclerotic plaques with low oxygen, the accumulation of lactate might reduce the anti-inflammatory effects of β-HB by shifting acetyl-CoA towards processes that promote inflammation (81). Advanced spatial multi-omics technologies, such as spatial transcriptomics and MALDI imaging mass spectrometry, are vital for understanding how tissue inflammation varies in atherosclerotic lesions (82). These technologies can identify unique metabolic and epigenetic signatures in various regions, enabling researchers to distinguish between pro-inflammatory monocytes in necrotic cores and reparative cells in fibrous caps (83, 84). We integrate dietary, genetic, and environmental data with a multi-omics human map to help uncover the complexities of multidimensional biological systems (85). This integration aims to develop predictive models that identify individual metabolic vulnerabilities. By addressing these challenges, we can formulate strategies to effectively modify tissue inflammation. This will change the management of atherosclerosis from simply managing risk factors to actively reshaping immune memory (22, 86).

## 4 Cross-organ regulation of immune memory: from bone marrow to therapeutic integration

### 4.1 Bone marrow as a hub of epigenetic inheritance

Tissue immunity is influenced by distant organs, which create a “training axis” that connects local and systemic immune responses (87). Bone marrow is a key center for systemic immune memory, and HSCs are essential for preserving epigenetic information (88). When exposed to chronic metabolic or inflammatory challenges, like high cholesterol levels or persistent cytokine exposure, HSCs undergo reprogramming that involves changes in DNA methylation and histone modifications (89). Under chronic metabolic/inflammatory stress relevant to atherosclerosis, bone-marrow progenitors and HSCs undergo durable reprogramming: Western diet in Ldlr^−/−^ mice elicits NLRP3-dependent epigenomic/transcriptomic remodeling of myeloid progenitors with heightened innate responses; peripheral ischemia in Apoe^−/−^ mice imposes epigenetic imprints in HSCs that propagate inflammation and accelerate atherosclerosis; conversely, enhancing cholesterol efflux (rHDL/LXR) or exercise restores HSPC quiescence and reduces inflammatory leukocyte output and plaque inflammation (37, 90–92). Epigenetic changes passed on to myeloid progenitors lead to monocytes that are ready for stronger inflammatory responses, even in the absence of ongoing triggers (93). This phenomenon is known as trained immunity (94). In studies using mouse models, HSCs from mice with high cholesterol produce monocytes that exhibit increased NLRP3 inflammasome activity (95), accelerating plaque progression in recipient animals. his systemic memory reveals the bone marrow’s critical role in sustaining vascular inflammation over time (96), thereby challenging the traditional view that atherosclerosis is strictly a localized condition.

### 4.2 Gut microbiota and SCFAs: orchestrating immune memory

The gut microbiota plays a crucial role in immune memory by producing metabolites, especially SCFAs such as butyrate, propionate, and acetate (97, 98). Butyrate is produced when bacteria ferment dietary fiber, and this compound plays a crucial role by inhibiting histone deacetylases (HDACs) in monocytes (99). Butyrate, a metabolite produced by microbiota, activates lncRNA lncLy6C, which in turn drives the differentiation of Ly6C(high) macrophages into Ly6C(int/neg) macrophages, mediated by the lncLy6C/C/EBPβ/Nr4A1 signaling axis (100). In the colonic lumen, it functions as a chemoprotective inhibitor of histone deacetylases and as an acetylation substrate for histone acetylases (101). Furthermore, SCFAs enhance mitochondrial biogenesis by activating PPARγ coactivator 1α (PGC-1α), which counteracts the glycolytic shift triggered by metabolic stressors such as oxLDL (102). On the other hand, dysbiosis, characterized by a decrease in SCFA-producing bacteria, can worsen trained immunity (103). This is particularly evident in models that mimic a Western diet, where increased gut permeability allows lipopolysaccharides (LPS) to enter the bloodstream, activating Toll-like receptor 4 (TLR4) and priming monocytes in a pro-inflammatory manner (104). The gut-vascular axis may serve as a promising target for adjusting immune memory, indicating that dietary changes or probiotic treatments could be helpful.

### 4.3 Hepatic cholesterol metabolism: stabilizing immune memory

The liver is essential for regulating the flexibility of monocytes by managing cholesterol metabolism and producing apolipoproteins (105). A key player in this process is apolipoprotein E (ApoE), primarily produced by hepatocytes in the liver (106, 107). ApoE plays a key role in cholesterol removal from monocytes by interacting with ABCA1 transporters. This interaction is critical for maintaining mitochondrial health and ensuring the proper function of SIRT1, a protein involved in cellular metabolism regulation (108). In their studies of ApoE-deficient mice, researchers found that cholesterol accumulation disrupts mitochondrial oxidative phosphorylation (109). This disruption compels cells to increasingly depend on glycolytic pathways for energy, stabilizes HIF-1α (a protein that enhances inflammation), and intensifies proinflammatory signals, ultimately leading to the destabilization of atherosclerotic plaques (110). Conversely, the introduction of liver X receptor (LXR) agonists increases the expression of ApoE (111), which aids in restoring cholesterol efflux and promotes an anti-inflammatory response via SIRT1-mediated deacetylation of NF-κB, a crucial regulator of inflammation (112). Furthermore, the liver affects systemic immune responses by signaling through bile acids (113). For example, FXR agonists such as obeticholic acid upregulate SIRT3 in monocytes, thereby enhancing mitochondrial deacetylation and oxidative metabolism (114). These insights reveal that the liver’s metabolic functions are crucial for regulating immune memory stability, thereby linking dietary lipids to vascular inflammation.

### 4.4 Integrated therapeutic strategies: targeting cross-organ networks

The interdependence of bone marrow, gut, and liver in shaping immune memory highlights the need for therapies that can target multiple organs at once (115). Combination treatment strategies include the use of PCSK9 inhibitors to lower the activity of the mevalonate pathway and SCFA-producing probiotics. These strategies work together to suppress pro-inflammatory T cells and encourage a more balanced immune response (116). Additionally, engineered nanoparticles provide a means for precise delivery (117, 118). For instance, bone marrow-targeting particles can deliver DNMT3A inhibitors to reverse the hypermethylation of CXCL2 in hematopoietic stem cells, while nanoparticles targeting the gut can directly release butyrate to support the colonic microbiota (119). Techniques for gene editing, such as CRISPR-dCas9 systems that are delivered with lipid nanoparticles, can create specific epigenetic changes in various organs (120), and introduce the APOE4 variant in pluripotent stem cells (121). New tools, including AI platforms that utilize multi-omics data, can further refine these approaches by predicting how individual patients may respond to treatments that affect multiple organs (122). However, challenges remain, including reducing off-target effects and defining safe parameters for epigenetic editing. By combining insights from hematology, microbiology, and hepatology, this comprehensive strategy could transform atherosclerosis management. It shifts the focus from isolated risk factors to a holistic understanding of immune memory engineering.

## 5 Therapeutic innovation and clinical translation: targeting trained immunity

### 5.1 Rewriting immune memory: epigenetic editing and small molecule therapies

By providing precise control over chromatin states, epigenetic editing technologies are transforming our ability to modulate trained immunity (123). One of the key advancements is the use of CRISPR-dCas9 systems (124, 125), which utilize CRISPR/dCas9-based epigenetic modifiers to reactivate the endogenous TERT gene in unstimulated T cells found in peripheral blood mononuclear cells (PBMCs) by rewiring the epigenetic marks of the TERT promoter (126). Preclinical studies have demonstrated that BET inhibitors, like DDO-8926, and HDAC inhibitors, such as entinostat, offer complementary approaches for managing inflammation (127, 128). BET inhibitors block BRD4, a protein that activates enhancers at pro-inflammatory sites (129), In contrast, HDAC inhibitors increase histone acetylation, which promotes the expression of anti-inflammatory genes (130). Although there have been advancements, several challenges persist, such as off-target effects that unintentionally silence tumor suppressor genes and issues with delivery efficiency. To address these concerns, we must improve cell-specific targeting. This is illustrated by the development of monocyte-targeted nanoparticles and systems that utilize exosomes for delivery.

### 5.2 Balancing metabolism and immunity: repurposing drugs for immune resilience

Metabolic modulators are gaining attention as therapies that fulfill two important roles: they address lipid and glucose dysregulation and reprogram immune memory (131, 132). PCSK9 inhibitors are well-known for lowering LDL cholesterol levels (133), but they also suppress the mevalonate pathway in monocytes (134). This suppression decreases the mTOR activation that requires geranylgeranylation, which helps maintain eTreg cells (135). When mTOR inhibitors and SIRT1 activators are combined, they balance glycolytic and oxidative metabolism, stabilizing T cell inflammation (136, 137). Researchers have demonstrated in studies with diabetic mouse models that this combination reduces plaque buildup by enhancing mitochondrial respiration (138). Similarly, FXR agonists improve cholesterol efflux and are involved in regulating lipid metabolism, which helps counter glycolytic inflammation (139, 140). These approaches underscore the promising potential of repurposing metabolic drugs for immunomodulation; however, further refinement is needed to determine the optimal dosing and timing for these therapies.

### 5.3 Personalized medicine: harnessing AI to predict and optimize treatments

The metabolic-vascular axis indicates that hyperglycemia may increase monocyte inflammatory responses through epigenetic modifications. However, the metabolic-vascular axis is complex and dynamic, making it challenging to fully understand its regulatory network using traditional experimental methods. Integrating multidimensional data to predict individual inflammatory phenotypes has become a significant challenge for clinical translation. AI-driven multi-omics integration technologies are revolutionizing tissue inflammation treatment by facilitating personalized predictions and designing targeted interventions (141). Multi-omics platforms, including single-cell ATAC-seq and metabolomics, generate extensive datasets that machine learning models can analyze to identify different immune states (142). For example, convolutional neural networks (CNNs) trained on chromatin accessibility profiles can predict enhancer-promoter interactions that play a role in proinflammatory trained immunity. This capability aids in selecting suitable CRISPR targets (143). Additionally, reinforcement learning frameworks help tailor treatment regimens by learning from patient responses to previous therapies (144). For example, in silico trials that simulate the use of PCSK9 inhibitors and SCFAs can help determine dosing schedules that improve plaque stability and lower toxicity (145). Convolutional neural networks (CNN) have been successfully applied to identify immune biomarkers in atherosclerosis. Han Zhang et al. constructed a deep learning model of convolutional neural network based on gene-immunity correlation, which achieved an AUC of 0.933, a sensitivity of 92.3%, and a specificity of 87.5% in an independent external test for diagnosing advanced plaque (146). Machine learning models are essential for integrating genetic, epigenetic, and clinical data to classify patients into distinct trained immunity subtypes, such as “hyperinflammatory” or “tolerogenic,” thus allowing for more targeted therapeutic approaches (147). However, challenges like ensuring that models are generalizable and addressing the diversity of data, especially for underrepresented populations, remain significant obstacles in this field.

## 6 Future directions: charting the next frontier in trained immunity research

Research on trained immunity in atherosclerosis has uncovered complex interactions between metabolic, epigenetic, and systemic regulatory networks. However, several critical questions remain that will influence future studies in this field. A significant area of research focuses on how immune memory is inherited across generations. High cholesterol levels or obesity may change germ cells in a way that increases the risk of inflammatory responses in offspring (148, 149). For example, studies involving mice have shown that maternal exposure to oxidized oxLDL causes DNA methylation at anti-inflammatory genes like IL-10 and TGF-β in oocytes, which leads to offspring monocytes that exhibit a lasting pro-inflammatory tendency (150, 151). In addition, factors from fathers, such as alterations in mitochondrial transfer RNAs in sperm due to Western diets, might also influence how immune memory is inherited by future generations (152). To fully understand these mechanisms, it is essential to conduct longitudinal studies involving human cohorts, combined with advanced multi-omics profiling. This approach will differentiate inherited epigenetic changes from environmental influences and guide interventions that break the cycle of cardiovascular risk across generations.

It is equally important to define the long-lasting duration of immune memory (153). Current therapies often neglect the timing of therapeutic interventions in the context of tissue inflammation. Early interventions in the early stages of plaque formation can change the epigenetic landscape. In contrast, plaques that have advanced to later stages often show persistent pro-inflammatory states (154). Researchers can identify the best times for intervention by using AI to analyze longitudinal multi-omics datasets, which combine data on chromatin accessibility, metabolite flow, and plaque imaging (155). For example, machine learning models trained on data from atherosclerotic mouse models and a single blood drop can diagnose and classify the severity of atherosclerosis. This indicates that biomarkers and vascular factors in the blood can be detected and are linked to the early stages of atherosclerosis development (156). Furthermore, targeted delivery systems like lipid nanoparticles, which are specifically designed to reach bone marrow and carry CRISPR/Cas9 protein, offer effective means to reverse maladaptive immune memory while minimizing systemic toxicity (157). However, to implement these strategies, it is essential to address the varying immune training conditions found within plaques. Hypoxic cores, rich in lactate and mitochondrial DNA fragments, may sustain pro-inflammatory trained immunity by stabilizing HIF-1 (158), While fibrous caps contain repairing monocytes that are influenced by AIM2 gradients (159, 160). Advanced spatial multi-omics technologies, including MIBI and spatial transcriptomics, will enable the mapping of distinct niches, which in turn will inform the development of localized therapies (161). For example, an injectable composite hydrogel (SFD/CS/ZIF-8@QCT) can target specific areas within plaques. This hydrogel contains quercetin-modified zeolitic imidazolate framework-8 (ZIF-8@QCT) and demonstrates excellent functions, including antibacterial properties and immunomodulation, which enhance therapeutic outcomes (162).

AI and quantum computing have great potential to speed up medical discoveries (163, 164). Using quantum-enabled simulations, researchers can investigate the complex interactions between epigenetic and metabolic factors and predict the outcomes of specific perturbation events. In clinical practice, AI platforms and single-cell RNA can categorize patients into different T cell immunity subtypes, such as hyperinflammatory, tolerogenic, or metabolically resistant, thereby facilitating the development of more personalized treatment plans (165, 166). To realize this vision, it is crucial to confront and resolve significant moral and logistical challenges. The high costs of CRISPR therapies may increase existing health disparities, emphasizing the importance of global collaboration to guarantee fair access to these advanced treatments (167). Regulatory agencies must balance promoting innovation with the need for caution, particularly regarding inheritable epigenetic modifications. This balance requires the creation of international guidelines to ensure safety and informed consent.

Ultimately, advancing this field requires collaboration among immunologists, computational biologists, ethicists, and clinicians to turn research on trained immunity from a scientific curiosity into effective therapies. The future of trained immunity research will transform atherosclerosis management and shed light on immune memory’s role in chronic diseases worldwide by emphasizing teamwork across disciplines, promoting open-data initiatives, and designing patient-centered research.

## 7 Conclusion

The discovery of trained immunity has significantly changed our understanding of atherosclerosis, framing it as a disorder related to dysregulated immune responses and metabolic memory. The trained immunity plasticity spectrum model defines trained immunity as a dynamic balance among pro-inflammatory, tolerogenic, and anti-inflammatory states, shaped by mTOR/AMPK signaling and histone modifications. The trained immunity-metabolism-vascular axis explains how metabolic disturbances can affect monocytes at the epigenetic level and change vascular environments through exosomal microRNAs and signals from mitochondrial stress. The cross-organ trained immunity framework underscores the critical regulation among the bone marrow, gut, and liver, illustrating that atherosclerosis is significantly influenced by their inter-organ communication. Furthermore, dynamic immune reprogramming strategies hold great promise for resetting harmful immune memories. These strategies include CRISPR-based epigenetic editing, therapies aimed at metabolism, and AI-driven precision approaches. These advancements challenge traditional reductionist views and lead to new therapies aimed at engineering immune memory instead of merely managing symptoms. Future initiatives should aim to apply these findings in clinical practice, use spatial multi-omics, and promote global equity to ensure that these innovations benefit diverse populations and ultimately transform cardiovascular disease prevention and treatment.
