# Supplementary material for: Trained immunity in atherosclerosis: plasticity, metabolic-vascular axis, and AI-driven precision remodeling
Source: Front Immunol. 2025 Oct 10;16:1669796. doi: 10.3389/fimmu.2025.1669796 (PMC12549631; doi:10.3389/fimmu.2025.1669796)
Supplement: Supplementary file 1 [file DataSheet1.pdf]

Here is a concise, editor-facing explanation of how the chosen algorithms—and their comparison—let AI refine pathway roles across immunity, metabolism, and plasticity, with references.

What we integrate. We assemble four data layers—innate immune states (monocytes/macrophages), metabolic context (oxLDL, β-HB, SCFAs; glycolysis/OXPHOS proxies), epigenetic marks (e.g., H3K4me3, H3 acetylation), and vascular phenotypes (endothelial adhesion, VSMC state, plaque readouts). These heterogeneous signals are harmonized in a heterogeneous knowledge graph and in matrices suitable for pathway activity scoring.[1]

How models work together.

1. Graph models (KG/GNN) propose missing but plausible links among metabolites, pathways, and cell types—useful for cross-domain assimilation[1].
2. Multimodal factor analysis (MOFA/MOFA+) compresses all signals into a few latent factors that track "trained-immunity intensity," enabling direct comparison across modalities[2].
3. We quantify pathway activity from expression using PROGENy (perturbation-derived footprints) and GSVA (sample-level gene-set variation), so outputs are pathway-level and biologically interpretable[3, 4].
4. Interpretable prediction (regularized models) then evaluates which features best predict plaque-related outcomes, with SHAP attributing importance to specific pathways/axes. Where temporal/interventional priors exist, NOTEARS helps propose causal directions.

How comparison refines pathways. We retain only pathways that (i) are highly ranked by ≥2 independent model families (graph + factor + predictor), and (ii) show stable attributions across resampling. This consensus avoids model-specific bias and yields a short, testable list.

What emerges (with biological anchors). The pipeline consistently prioritizes:
AMPK–SIRT1–HDAC axis**:** higher AMPK/SIRT1 with lower HDAC activity aligns with increased H3 acetylation in innate cells and lower NLRP3 readiness—consistent with β-HB acting as an endogenous class-I HDAC inhibitor[5].
β-HB–GPR109A–NLRP3 axis: graph and predictive models converge on ketone-receptor signaling that dampens inflammasome activity; this matches human ex vivo suppression of NLRP3-dependent IL-1β/IL-18 and $ApoE^{-}/^{-}$ in-vivo plaque mitigation by oral 3-HB[6, 7].
These AI-refined axes sit on an atherosclerosis-specific trained-immunity backdrop (e.g., oxLDL-induced H3K4me3 memory in monocytes; Western diet–driven, NLRP3-dependent training in $Ldlr^{-}/^{-}$ mice), closing the loop from computation to mechanism[8, 9].

References：

1. Himmelstein DS, Lizee A, Hessler C, Brueggeman L, Chen SL, Hadley D, Green A, Khankhanian P, Baranzini SE. Systematic integration of biomedical knowledge prioritizes drugs for repurposing. *Elife* 2017;**6**.
2. Argelaguet R, Velten B, Arnol D, Dietrich S, Zenz T, Marioni JC, Buettner F, Huber W, Stegle O. Multi-

Omics Factor Analysis-a framework for unsupervised integration of multi-omics data sets. *Mol Syst Biol* 2018;**14**:e8124.

3. Schubert M, Klinger B, Klünemann M, Sieber A, Uhlitz F, Sauer S, Garnett MJ, Blüthgen N, Saez-Rodriguez J. Perturbation-response genes reveal signaling footprints in cancer gene expression. *Nat Commun* 2018;**9**:20.
4. Hänzelmann S, Castelo R, Guinney J. GSVA: gene set variation analysis for microarray and RNA-seq data. *BMC Bioinformatics* 2013;**14**:7.
5. Shimazu T, Hirschey MD, Newman J, He W, Shirakawa K, Le Moan N, Grueter CA, Lim H, Saunders LR, Stevens RD, Newgard CB, Farese RV, Jr., de Cabo R, Ulrich S, Akassoglou K, Verdin E. Suppression of oxidative stress by β-hydroxybutyrate, an endogenous histone deacetylase inhibitor. *Science* 2013;**339**:211-214.
6. Youm YH, Nguyen KY, Grant RW, Goldberg EL, Bodogai M, Kim D, D'Agostino D, Planavsky N, Lupfer C, Kanneganti TD, Kang S, Horvath TL, Fahmy TM, Crawford PA, Biragyn A, Alnemri E, Dixit VD. The ketone metabolite β-hydroxybutyrate blocks NLRP3 inflammasome-mediated inflammatory disease. *Nat Med* 2015;**21**:263-269.
7. Zhang SJ, Li ZH, Zhang YD, Chen J, Li Y, Wu FQ, Wang W, Cui ZJ, Chen GQ. Ketone Body 3-Hydroxybutyrate Ameliorates Atherosclerosis via Receptor Gpr109a-Mediated Calcium Influx. *Adv Sci (Weinh)* 2021;**8**:2003410.
8. Bekkering S, Quintin J, Joosten LA, van der Meer JW, Netea MG, Riksen NP. Oxidized low-density lipoprotein induces long-term proinflammatory cytokine production and foam cell formation via epigenetic reprogramming of monocytes. *Arterioscler Thromb Vasc Biol* 2014;**34**:1731-1738.
9. Christ A, Günther P, Lauterbach MAR, Duewell P, Biswas D, Pelka K, Scholz CJ, Oosting M, Haendler K, Baßler K, Klee K, Schulte-Schrepping J, Ulas T, Moorlag S, Kumar V, Park MH, Joosten LAB, Groh LA, Riksen NP, Espevik T, Schlitzer A, Li Y, Fitzgerald ML, Netea MG, Schultze JL, Latz E. Western Diet Triggers NLRP3-Dependent Innate Immune Reprogramming. *Cell* 2018;**172**:162-175.e114.
